# Increased Expression of INHBA Is Correlated With Poor Prognosis and High Immune Infiltrating Level in Breast Cancer

**DOI:** 10.3389/fbinf.2022.729902

**Published:** 2022-03-24

**Authors:** Zeying Yu, Li Cheng, Xinlian Liu, Lushun Zhang, Hui Cao

**Affiliations:** ^1^ Department of Pathogenic Biology, School of Basic Medical Science, Chengdu Medical College, Chengdu, China; ^2^ School of Basic Medical Science, Chengdu Medical College, Chengdu, China; ^3^ Department of Pathology and Pathophysiology, School of Basic Medical Science, Chengdu Medical College, Chengdu, China

**Keywords:** breast cancer, macrophages, prognosis, INHBA, CAFs

## Abstract

**Background:** Inhibin, beta A (INHBA) is a member of the transforming growth factor-β superfamily and is associated with carcinogenesis and cancer progression in several types of human cancers. However, its significance in breast cancer has not been evaluated. Here, we investigated the prognostic value of INHBA and its correlation with tumor-infiltration immune cells in the microenvironment of breast cancer.

**Methods:** In this study, we analyzed the INHBA expression profile in the Oncomine database and Tumor Immune Estimation Resource 2.0 (TIMER2.0) site. Using Breast Cancer Gene-Expression Miner (bc-GenExMiner v4.7) tool and the UALCAN cancer database, we further evaluated the correlation of INHBA expression with clinicopathological factors in breast cancer. Then, we assessed the clinical prognostic value of INHBA using Kaplan–Meier Plotter and the PrognoScan databases. The correlations between INHBA and tumor-infiltrating immune cells were investigated *via* TIMER2.0. In addition, correlations between INHBA expression and gene markers of immune infiltrates were analyzed by TIMER2.0 and Gene Expression Profiling Interactive Analysis 2.

**Results:** Compared with the level in normal tissues, the INHBA mRNA expression was upregulated in different subtypes of breast cancer, and its expression was positively correlated with progesterone receptor, human epidermal growth factor receptor-2 status, and PAM50 subtypes but negatively related to age and basal-like status. The INHBA protein was also highly expressed in primary breast cancer and closely related to the pathological stage. Patients with high INHBA expression levels showed worse overall survival, relapse-free survival, and distant metastasis-free survival. Also, high INHBA expression was significantly associated with worse overall survival and relapse-free survival in positive lymph nodes. Of interest, INHBA expression was negatively correlated with infiltrating levels of activated NK cells, NKT, and CD4^+^ T cells but was positively correlated with tumor infiltration of CD8^+^ T cells, neutrophils, especially macrophages and cancer-associated fibroblasts. Moreover, INHBA expression showed strong correlations with various markers of monocytes/macrophages and cancer-associated fibroblasts.

**Conclusion:** High INHBA expression is correlated with poor prognosis and the infiltration of immune cells in the tumor microenvironment. These findings suggest that INHBA may be involved in immune escape and can serve as a potential biomarker of prognosis and tumor-infiltrating immune cells.

## Introduction

Breast cancer ranks first among the global top 10 cancers and remains the leading cause of female cancer-related deaths worldwide in 2020. In China, the incidence and mortality of breast cancer rank first and fourth among women, respectively (Sung et al., 2021). Still, patients with breast cancer that has already metastasized to other sites at the time of diagnosis have the worst prognosis. The mutual interactions between cancer cells and the tumor microenvironment play important roles in cancer progression toward metastasis (Mantovani, 2009). Growing research has suggested that immune infiltrates in the tumor microenvironment, such as tumor-associated macrophages (TAMs), tumor-infiltrating lymphocytes, and caner-associated fibroblasts (CAFs), are related to the patients' survival (Iglesia et al., 2016; Denkert et al., 2018; Chen et al., 2021). Also, immunotherapy as the most promising method has been widely applied for cancer treatment (Emens, 2018). Therefore, exploring the precise prognostic correlation of immune infiltrates in breast cancer and finding new immune-related therapeutic targets are urgent for early diagnosis and treatment of breast cancer.

Inhibin, beta A (INHBA), which belongs to a member of the transforming growth factor-β (TGF-β) superfamily, can form both activin A by homo-dimerizing and inhibin by hetero-dimerizing with inhibin (Murata et al., 1988; Brown et al., 2000). Activin A and inhibin are two closely related glycoproteins with opposing biological effects and extensively involved in many processes of organismal and embryonic development, as well as carcinogenesis and cancer progression (Vale et al., 1988; Chen et al., 2006). As the subunit of activin A and inhibin, INHBA was reported highly expressed in multiple human cancers, including gastric cancer (Wang et al., 2012; Oshima et al., 2014), colorectal cancer (Okano et al., 2013), esophageal adenocarcinoma (Seder et al., 2009a), urothelial carcinoma (Lee et al., 2015), and lung cancer (Seder et al., 2009b). The high levels of INHBA promoted cancer invasion and metastasis and were associated with poor survival of patients. However, its clinical significance and prognostic effects in breast cancer have not been systemically evaluated.

Here, we comprehensively evaluated INHBA expression and the association between INHBA levels and breast cancer patients' prognosis in public databases such as Oncomine, Kaplan–Meier plotter, and PrognoScan. Then, we analyzed the correlation of INHBA expression with immune infiltrating levels in the tumor microenvironment using Tumor Immune Estimation Resource 2.0 (TIMER2.0) and Gene Expression Profiling Interactive Analysis 2 (GEPIA2). Our findings reveal that high expression of INHBA was an adverse prognostic biomarker for breast cancer and provide an underlying mechanism between INHBA and tumor–immune interactions.

## Materials and Methods

### Oncomine Platform

INHBA mRNA expression levels in different types of cancers were analyzed in the Oncomine platform (https://www.oncomine.org/resource/login.html) (Rhodes et al., 2007), which has a powerful set of analysis functions that compute gene expression signatures, clusters, and gene-set modules, automatically extracting biological insights from the data. The threshold settings were determined according to the following values: *p*-value of 0.001, fold change of 2.0, gene ranking of top 10%, and data type from mRNA.

### Breast Cancer Gene-Expression Miner v4.7

The correlation of INHBA mRNA expression with clinicopathological factors in breast cancer was analyzed using the Breast Cancer Gene-Expression Miner v4.7 (bc-GenExMiner v4.7) (http://bcgenex.ico.unicancer.fr), a statistical mining tool of published annotated breast cancer transcriptomic data (Jezequel et al., 2013). INHBA expression analysis was performed in the “expression” module, and the used data were from RNA-seq.

### UALCAN Cancer Database

UALCAN (http://ualcan.path.uab.edu) is a comprehensive web resource to analyze cancer OMICS data. Now, UALCAN can provide protein expression analysis based on data from the Clinical Proteomic Tumor Analysis Consortium Confirmatory/Discovery dataset (Chandrashekar et al., 2017). In this study, we evaluated INHBA protein expression and its relationship with clinicopathological factors in breast cancer by Clinical Proteomic Tumor Analysis Consortium analysis.

### Kaplan–Meier Plotter Database

The clinical prognostic value of INHBA mRNA and protein expression in breast cancer was evaluated using Kaplan–Meier plotter (http://kmplot.com/analysis), a powerful online database of gene expression data and clinical data from 21 cancer types, including breast (*n* = 6,234), ovarian (*n* = 2,190), lung (*n* = 3,452), and gastric (*n* = 1,440) cancers (Gyorffy et al., 2010). Log-rank *p*-values and the hazard ratios (HRs) with 95% confidence intervals (CIs) were also computed on the webpage.

### PrognoScan Database

PrognoScan (http://dna00.bio.kyutech.ac.jp/PrognoScan/index.html) is a powerful database for meta-analysis of the prognostic value of genes (Mizuno et al., 2009). It provides the correlation between gene expression and patient prognoses such as overall survival (OS), disease-free survival, and distant metastasis-free survival (DMFS) across a large number of public cancer microarray datasets. The PrognoScan database assessed the relationship between INHBA expression and survival in breast cancer. Cox *p*-values and HRs with 95% confidence intervals were also calculated automatically. The threshold was set as *p*-value < 0.05.

### Tumor Immune Estimation Resource 2.0 Database

TIMER2.0 (http://timer.cistrome.org) is a comprehensive web resource for the systematic analysis of tumor-infiltrating immune cells across diverse cancers (Li T. et al., 2020). This version of the webserver provides immune infiltrates' abundances estimated by TIMER, CIBERSORT, quanTIseq, xCell, MCP-counter, and EPIC algorithms. The “Gene_DE” module was used to study the differential expression of INHBA between tumor and adjacent normal tissues across all The Cancer Genome Atlas (TCGA) tumors. Distributions of gene expression levels were displayed using box plots. The statistical significance computed by the Wilcoxon test was annotated by the number of stars (*p < 0.05; **p < 0.01; ***p < 0.001). We explored the correlation of INHBA expression with immune infiltration level in BRCA (breast invasive carcinoma) and the subtypes in the “Gene” module. Tumor purity is a major confounding factor in this analysis, and it is recommended to adjust purity for the association analysis. Especially for methods such as EPIC, which provides cell fractions referred to as total cells, there is no need to adjust purity for the association analysis using the estimations from EPIC. We displayed the gene expression levels against tumor purity on the leftmost panel. Furthermore, we assessed the relationships between INBHA expression and gene markers of tumor-infiltrating immune cells *via* the “Gene_Corr” module. A total of 86 related gene markers from the Cell Marker database (http://biocc.hrbmu.edu.cn/Cell Marker/) were used for this study. The gene expression level was displayed with log2 TPM. Spearman's correlation coefficient and the estimated statistical significance were also available (*p < 0.01; **p < 0.001; ***p < 0.0001).

### Gene Expression Profiling Interactive Analysis 2 Database

GEPIA2 (http://gepia2.cancer-pku.cn/#index) is a new tool to analyze RNA sequencing expression data from TCGA and the Genotype-Tissue Expression datasets (Tang et al., 2017). To further confirm gene expression correlation analysis in TIMER2.0, we performed correlation analysis between INHBA and related genes and markers of monocytes/macrophages and CAFs in GEPIA2. The Spearman's correlation coefficient was used to evaluate the correlation of gene expression (*p < 0.01; **p < 0.001; ***p < 0.0001).

### Statistical Analysis

Gene expression results obtained from Oncomine were displayed with *p*-values, *t*-test, fold change, and gene rank. A Welch's test and Dunnett–Tukey–Kramer's tests were used to investigate the significance of the correlation of INHBA expression with clinicopathological features in breast cancer. The survival results from PrognoScan and Kaplan–Meier plotter were shown with HRs with 95% confidence intervals and *P* or Cox *p*-value based on a log-rank test. Spearman's correlation evaluated the correlation of gene expression. *p* < 0.05 was considered statistically significant, and the strength of correlation was referred to the following guide for the Rho value: 0.00–0.19 (very weak), 0.20–0.39 (weak), 0.40–0.59 (moderate), 0.60–0.79(strong), and 0.80–1.0 (very strong).

## Results

### INHBA Expression Level in Breast Cancer

**FIGURE 1:**
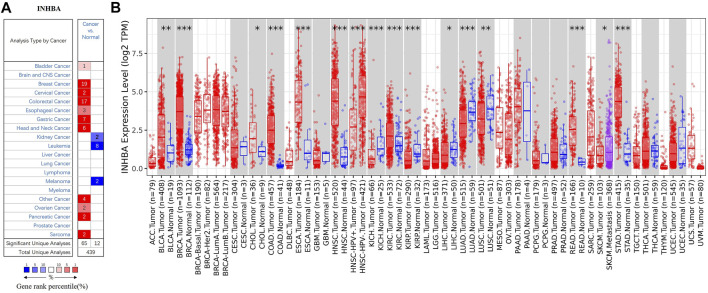
mRNA expression of INHBA in different types of human cancers. **(A)** Increased or decreased expression of INHBA in datasets of different cancers compared with normal tissues in Oncomine database. Number in each cell is amount of datasets. **(B)** Human INHBA expression of different tumor types from TCGA database was determined by TIMER2.0 (*p < 0.05, **p < 0.01, ***p < 0.001).

**FIGURE 2:**
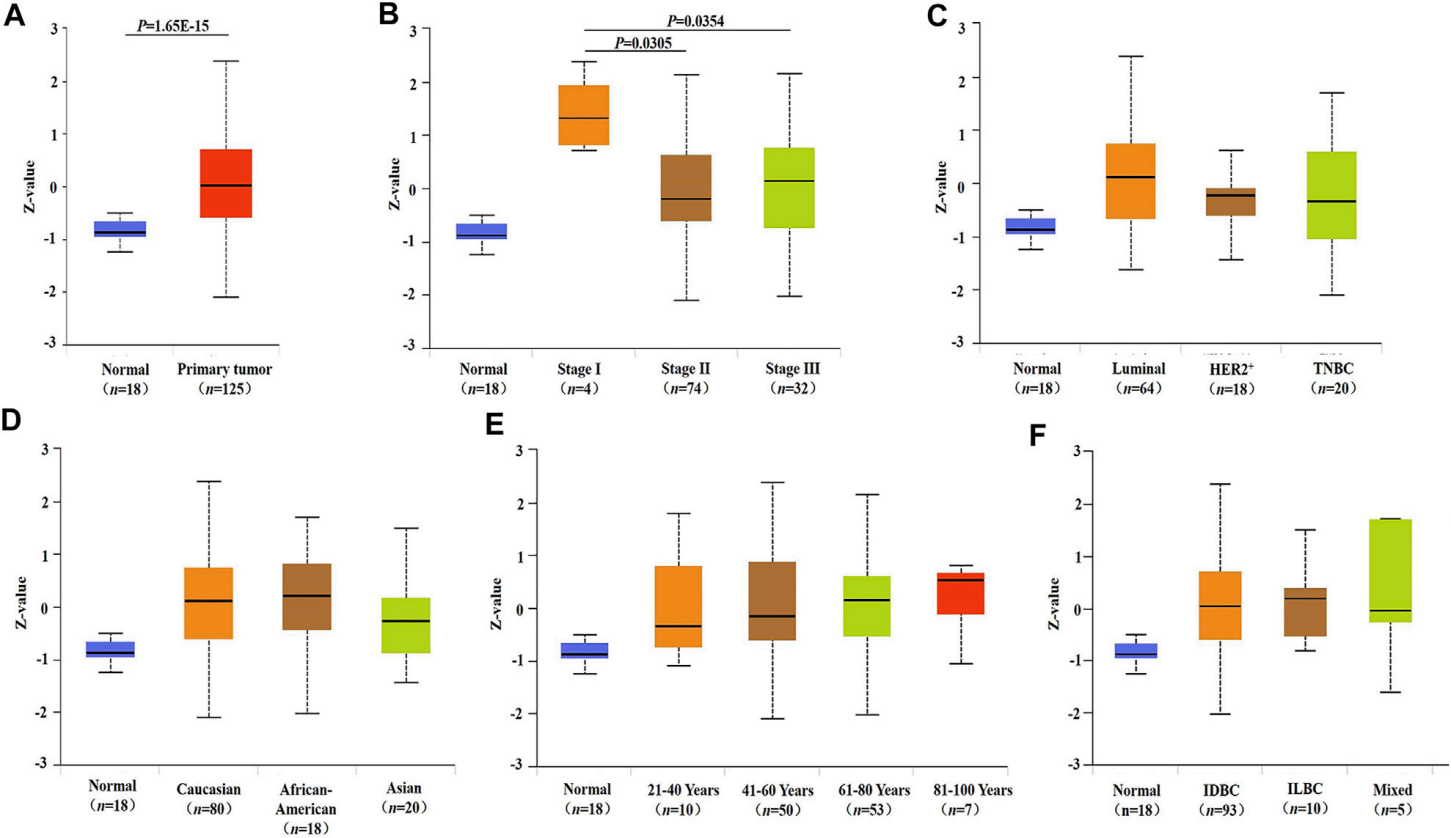
INHBA protein expression and its relationship with clinicopathological factors in breast cancer. **(A)** Protein expression of INHBA in primary breast cancer and normal tissues analyzed by UALCAN. **(B–F)** Relationship between protein expression of INHBA and clinicopathological features in breast cancer analyzed by UALCAN. Z-values represent standard deviations from median across samples for given cancer type. Log2 Spectral count ratio values from Clinical Proteomic Tumor Analysis Consortium were first normalized within each sample profile, then normalized across samples. IDBC, Invasive ductal breast carcinoma; ILDC, Invasive lobar breast carcinoma; Mixed, Mixed lobular and ductal breast carcinoma.

The mRNA expression of INHBA in different types of human cancer was analyzed using the Oncomine database. The results showed that INHBA mRNA expression was upregulated in bladder, breast, cervical, colorectal, esophageal, gastric, head and neck, ovarian, and pancreatic cancers and sarcoma with respect to normal tissues, whereas INHBA was downregulated in kidney cancer, leukemia, and melanoma. Notably, INHBA mRNA expression was significantly higher in 19 breast cancer datasets than in normal breast tissues (Figure 1A). The detailed data of INHBA mRNA expression in different subtypes of breast cancer are shown in Supplementary Table S1. Next, human INHBA expression of different tumor types from TCGA was further determined by TIMER2.0 (Figure 1B). The analysis revealed that INHBA was highly expressed in BLCA (bladder urothelial carcinoma), BRCA (breast invasive carcinoma), CHOL (cholangiocarcinoma), COAD (colon adenocarcinoma), ESCA (esophageal carcinoma), HNSC (head and neck squamous cell carcinoma), KIRC (kidney renal clear cell carcinoma), READ (rectum adenocarcinoma), and STAD (stomach adenocarcinoma) relative to normal adjacent tissues, whereas INHBA expression was decreased in KICH (kidney chromophobe), KIRP (kidney renal papillary cell carcinoma), LIHC (liver hepatocellular carcinoma), LUAD (lung adenocarcinoma), and LUSC (lung squamous cell carcinoma). Consistently, we observed that INHBA mRNA expression was significantly elevated in BRCA and the subtypes (BRCA-Basal, BRCA-Her2, BRCA-LumA, and BRCA-LumB) compared with adjacent normal tissues. Besides, higher protein expression of INHBA was also found in primary breast cancer compared with normal tissues in the UALCAN cancer database (Figure 2A). Taken together, the data confirmed that the *INHBA* gene was upregulated in breast cancer compared with normal samples.

### Correlation of INHBA Expression and Clinicopathological Factors in Breast Cancer

We compared INHBA mRNA expression among groups of breast cancer patients according to different clinicopathological factors in the bc-GenExMiner v4.7 database. We found that the mRNA expression of INHBA was significantly higher in the ≤51 compared with >51 years group. Breast cancer patients with positive progesterone receptor and human epidermal growth factor receptor-2 showed an increased level of INHBA mRNA expression. INHBA was elevated in the non-basal-like subtype with respect to the basal-like subtype. Additionally, PAM50 subtypes were associated with INHBA mRNA expression. INHBA was strongly upregulated in Basal, Her2, LumA, and LumB subtypes compared with normal breast-like subtypes. The detailed results are shown in Table 1. Next, we analyzed the relationships between the protein expression of INHBA and clinicopathological features in breast cancer using the UALCAN cancer database. We found that INHBA protein expression was significantly higher in the stage I group compared with the stage II and III groups (Figure 2B). However, its protein expression was not related to major subclass (Figure 2C), patient's race (Figure 2D), age (Figure 2E), and tumor histology (Figure 2F).

**TABLE 1:** Correlation of INHBA mRNA expression and clinicopathological factors in breast cancer (bc-GenExMiner v4.7).

Parameters	*N*	INHBA mRNA	*p*-value
Age (years)			
≤51	1,099	↑	**0.0025**
>51	3,208	Ref.	
ER			
Negative	551	Ref.	0.6624
Positive	3,911	—	
PR			
Negative	828	Ref.	**0.0200**
Positive	3,498	↑	
HER2			
Negative	3,582	Ref.	**<0.0001**
Positive	661	↑	
Nodal status			
Negative	2,415	Ref.	0.0686
Positive	1,646	—	
Basal-like status			
Non-basal-like	3,837	↑	**0.0291**
Basal-like	832	Ref.	
Triple-negative status			
Non-triple-negative	4,119	Ref.	0.1365
Triple-negative	317	—	
PAM50 subtypes			
Normal breast-like	639	Ref.	**<0.0001**
Basal	832	↑	
Her2	736	↑	
LumA	1,433	↑	
LumB	1,029	↑	

Bold value indicate *p* < 0.05.

### Prognostic Value of INHBA in Breast Cancer

**FIGURE 3:**
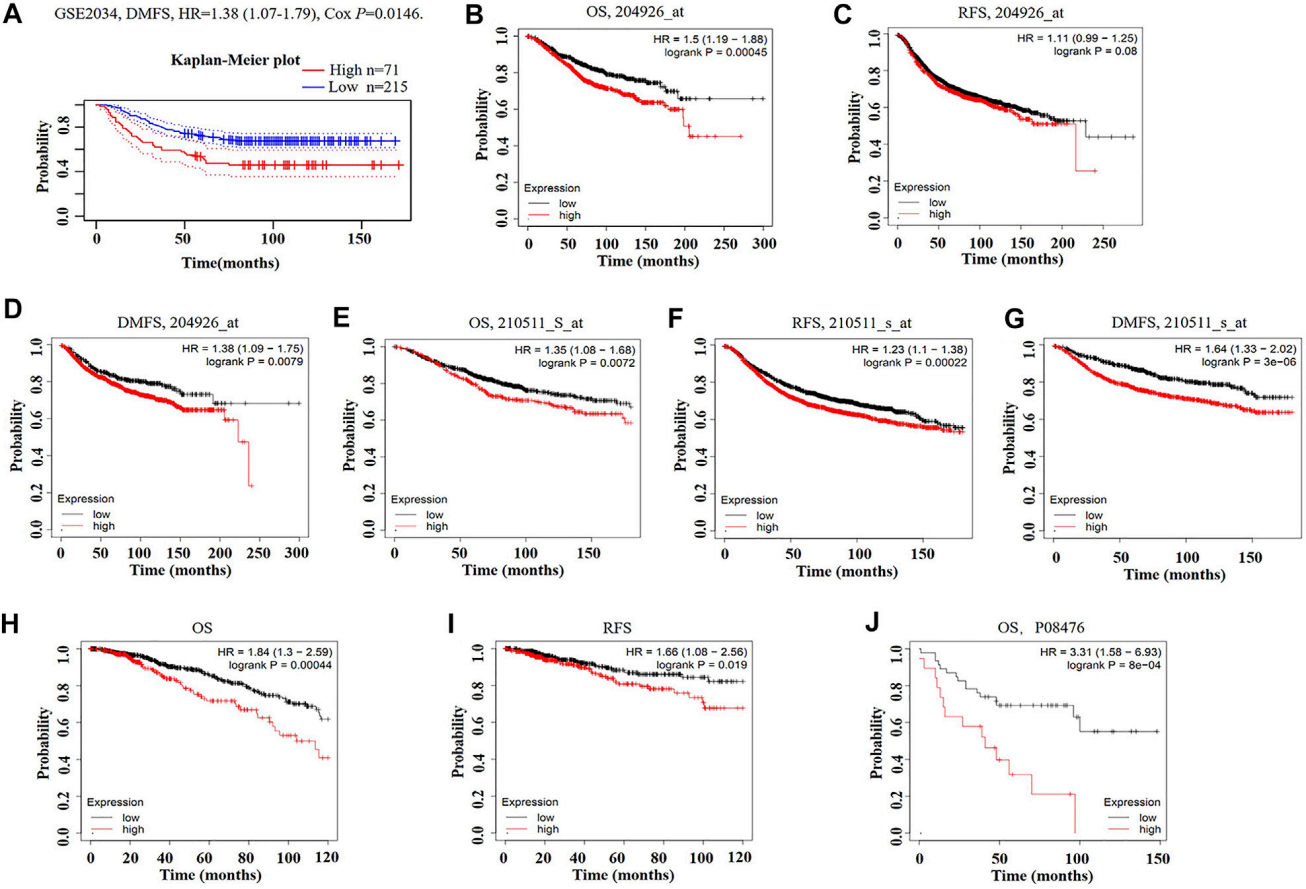
Kaplan–Meier survival curves comparing high and low expression of INHBA in breast cancer in PrognoScan **(A)** and Kaplan–Meier plotter databases **(B–J)**. **(A)** Survival curves of DMFS in breast cancer cohort [GSE 2034 (*n* = 286)]. **(B–D)** OS, RFS, and DMFS survival curves of breast cancer using probe set 204926_at (*n* = 1,402, *n* = 3,951, *n* = 1,746). **(E–G)** OS, RFS, and DMFS survival curves of breast cancer using probe set 210511_s_at (*n* = 1,402, *n* = 3,951, *n* = 1,746). **(H, I)** OS and RFS survival curves of breast cancer using pan-cancer RNA-seq (*n* = 1,089, *n* = 947). **(J)** Relationship between INHBA protein expression and OS in breast cancer (*n* = 65). OS, overall survival; RFS, relapse-free survival; DMFS, distant metastasis-free survival.

We then analyzed the prognostic potential of INHBA gene in breast cancer patients. The PrognoScan database showed that INHBA mRNA overexpression was significantly associated with inferior DMFS {[HR (95%CI) = 1.38 (1.07–1.79)], *p* = 0.0146} in GSE2034 datasheet (Figure 3A). The Kaplan–Meier plotter revealed that breast cancer patients with upregulated INHBA demonstrated worse prognosis using probe set 20496_at [Figures 3B–D; OS HR (95%CI) = 1.5 (1.19–1.88), *p* = 0.00045; RFS HR (95%CI) = 1.11 (0.99–1.25), *p* = 0.08; DMFS HR (95%CI) = 1.38 (1.09–1.75), *p* = 0.0079]. Similarly, INHBA expression was significantly correlated with worse prognosis in breast cancer patients using probe set 210511_s_at [Figures 3E–G; OS HR (95%CI) = 1.35 (1.08–1.68), *p* = 0.0072; RFS HR (95%CI) = 1.23 (1.1–1.38), *p* = 0.00022; DMFS HR (95%CI) = 1.64 (1.33–2.02), *P* = 3e-06]. Furthermore, to further investigate the role of INHBA in breast cancer prognosis, we verified that INHBA was negatively correlated with OS [HR (95%CI) = 1.84 (1.3–2.59), *p* = 0.00044] and RFS [HR (95%CI) = 1.66 (1.08–2.56), *p* = 0.019] using pan-cancer RNA-seq data from the Kaplan–Meier plotter database (Figures 3H,I). In addition, we also detected that the high INHBA protein expression was significantly related to the worse OS in Kaplan–Meier plotter database (Figure 3J). Conclusively, it is believable that high INHBA expression could be a risk factor for a poor prognosis in breast cancer patients.

### High INHBA Expression Affects the Prognosis of Breast Cancer Patients With Lymph Node Metastasis

To further understand the potential mechanisms of INHBA expression in cancer, we analyzed the correlation of INHBA expression and clinical prognosis in breast cancer patients with different clinicopathological factors by Kaplan–Meier plotter (Table 2). Overexpression of INHBA mRNA was associated with worse OS and RFS in the basal subtype of breast cancer (*p* < 0.05). It is worth noting that high mRNA expression of INHBA was significantly associated with worse OS and RFS in positive lymph nodes [OS HR (95%CI) = 1.95 (1.32–2.89), *p* = 0.0006; RFS HR (95%CI) = 1.51 (1.24–1.84), *p* = 4.1e-05] as well as in grade 3 of breast cancer patients [OS HR (95%CI) = 1.60 (1.13–2.28), *p* = 0.0082; RFS HR (95%CI) = 1.41 (1.12–1.77), *p* = 0.0034]. These data imply that INHBA expression level can affect the outcomes of breast cancer patients with lymphatic metastasis.

**TABLE 2:** Correlation of INHBA mRNA expression and clinical prognosis in breast cancer with different clinicopathological factors by Kaplan–Meier plotter.

Reference	Overall survival (*n* = 1,402)			Relapse free survival (*n* = 3,955)		
	*N*	Hazard ratio	*p*-value	*N*	Hazard ratio	*p*-value
ER						
Negative	251	1.69 (1.03–2.78)	0.0363	801	1.19 (0.95–1.49)	0.1322
Positive	548	1.34 (0.90–1.98)	0.1460	2,061	1.19 (1.00–1.42)	0.0527
PR						
Negative	89	0.53 (0.21–1.33)	0.1670	549	1.37 (0.96–1.94)	0.0813
Positive	83	0.46 (0.11–1.85)	0.2601	589	0.85 (0.60–1.21)	0.3759
HER2						
Negative	130	3.95 (0.92–16.99)	**0.0467**	800	1.27 (0.97–1.67)	0.0798
Positive	129	0.63 (0.31–1.29)	0.2002	252	1.25 (0.76–2.03)	0.3798
PAM50 subtypes						
Basal	241	1.78 (1.07–2.96)	**0.0255**	618	1.40 (1.01–1.68)	**0.0435**
Her2	117	1.56 (0.79–3.06)	0.1971	251	1.26 (0.80–1.98)	0.3227
LumA	611	1.58 (1.03–2.44)	**0.0361**	1,933	1.07 (0.90–1.27)	0.4163
LumB	433	1.90 (1.23–2.92)	**0.0032**	1,149	1.15 (0.95–1.40)	0.1434
Nodal status						
Negative	594	0.61 (0.41–0.91)	**0.0131**	2,020	0.89 (0.73–1.08)	0.2326
Positive	313	1.95 (1.32–2.89)	**0.0006**	1,133	1.51 (1.24–1.84)	**4.1e-05**
Grade						
1	161	—	—	345	1.83 (0.98–3.40)	0.054
2	387	0.71 (0.46–1.09)	0.1168	901	0.68 (0.53–0.87)	**0.0021**
3	503	1.60 (1.13–2.28)	**0.0082**	903	1.41 (1.12–1.77)	**0.0034**
TP53 status						
Mutated	111	3.51 (1.05–11.75)	**0.0297**	188	1.47 (0.90–2.42)	0.1229
Wild type	187	1.50 (0.77–2.93)	0.2263	273	1.34 (0.87–2.06)	0.1786

Bold value indicate *p* < 0.05.

### INHBA Expression Levels Are Associated With Tumor-Infiltrating Immune Cells in Breast Cancer

**FIGURE 4:**
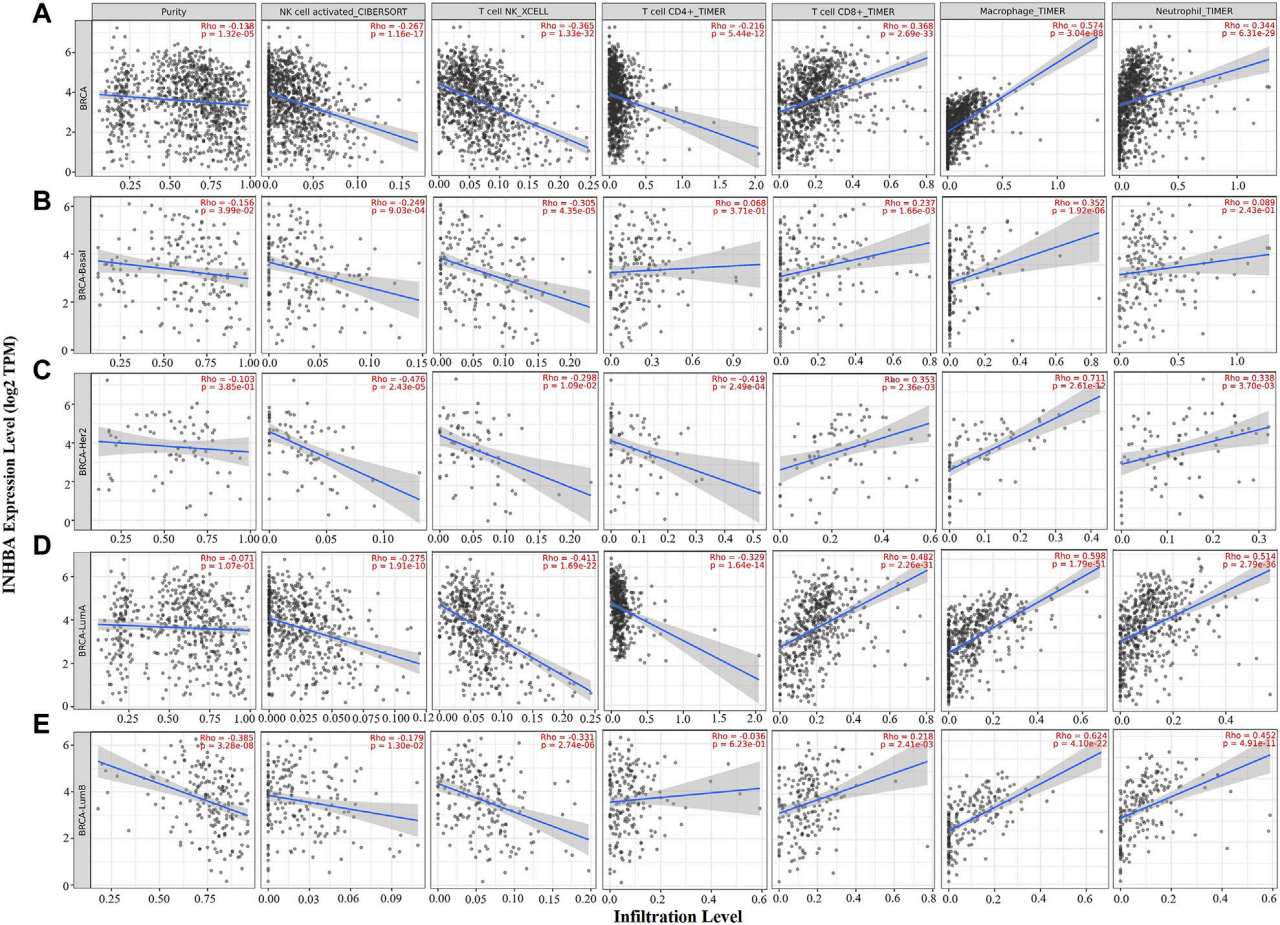
Correlation of INHBA expression with immune infiltration level in BRCA (breast invasive carcinoma) and subtypes (BRCA-Basal, BRCA-Her2, BRCA-LumA, and BRCA-LumB) analyzed by TIMER2.0. **(A,C,D)** INHBA expression was significantly negatively related to activated NK, NKT, and CD4^+^ T cell infiltrating levels and had significantly positive correlations with infiltrating levels of CD8^+^ T cells, macrophages, and neutrophils in BRCA (*n* = 1,100), BRCA-Her2 (*n* = 82), and BRCA-LumA (*n* = 568). **(B)** INHBA expression was significantly negatively related to activated NK and NKT cell infiltrating levels and had significantly positive correlations with infiltrating levels of CD8^+^ T cells and macrophages in BRCA-Basal (*n* = 191). **(E)** INHBA expression was significantly negatively related to activated NK and NKT cell infiltrating levels and had significant positive correlations with infiltrating levels of CD8^+^ T cells, macrophages, and neutrophils in BRCA-LumB (*n* = 219).

**FIGURE 5:**
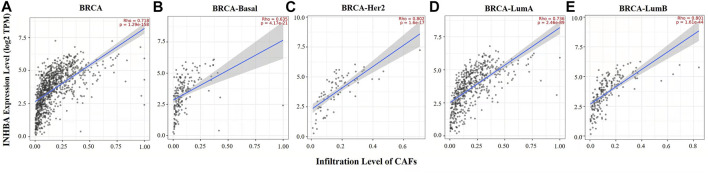
Correlation between INHBA expression and abundance of CAFs in BRCA and subtypes (BRCA-Basal, BRCA-Her2, BRCA-LumA, and BRCA-LumB) analyzed by TIMER2.0. **(A–E)** INHBA expression had strongly positive correlations with CAF infiltration in BRCA (*n* = 1,100), BRCA-Basal, BRCA-Her2 (*n* = 82), BRCA-LumA (*n* = 568), and BRCA-LumB (*n* = 219).

Immune cell infiltrates play an important role throughout breast carcinogenesis and progression (de la Cruz-Merino et al., 2013; Goff and Danforth, 2021). Thus, we analyzed the correlations of INHBA expression with immune infiltration levels in BRCA (breast invasive carcinoma) and the subtypes (BRCA-Basal, BRCA-Her2, BRCA-LumA, and BRCA-LumB) by TIMER2.0. In BRCA (*n* = 1,100), INHBA expression had significantly negative correlations with infiltrating levels of activated NK cells (Rho = −0.267, *p* = 1.16e-17), NKT cells (Rho = −0.365, *p* = 1.33e-32), and CD4^+^ T cells (Rho = −0.216, *p* = 5.44e-12) and had significantly positive correlations with infiltrating levels of CD8^+^ T cells (Rho = 0.368, *p* = 2.69e-33), macrophages (Rho = 0.574, *p* = 3.04e-88), and neutrophils (Rho = 0.344, *p* = 6.31e-29) (Figure 4A). However, the correlations were not all the same in different breast cancer subtypes. In BRCA-Basal (*n* = 191), INHBA expression was negatively related to activated NK cell (Rho = -0.249, *p* = 9.03e-4) and NKT cell (Rho = -0.305, *p* = 4.35e-5) infiltration levels and showed positive correlations with infiltrating levels of CD8^+^ T cells (Rho = 0.237, *p* = 1.66e-3) and macrophages (Rho = 0.352, *p* = 1.92e-6) (Figure 4B). In BRCA-Her2 (*n* = 82), INHBA expression had significantly negative correlations with infiltrating levels of activated NK cells (Rho = -0.476, *p* = 2.43e-5), NKT cells (Rho = -0.298, *p* = 1.09e-2), and CD4^+^ T cells (Rho = -0.419, *p* = 2.49e-4) and had significantly positive correlations with infiltrating levels of CD8^+^ T cells (Rho = 0.353, *p* = 2.36e-3), macrophages (Rho = 0.711, *p* = 2.61e-12), and neutrophils (Rho = 0.338, *p* = 3.70e-3) (Figure 4C). In BRCA-LumA (*n* = 568), INHBA expression had significant positive correlations with infiltrating levels of activated NK cells (Rho = -0.275, *p* = 1.91e-10), NKT cells (Rho = -0.411, *p* = 1.69e-22), and CD4^+^ T cells (Rho = -0.329, *p* = 1.64e-14) and had significantly positive correlations with infiltrating levels of CD8^+^ T cells (Rho = 0.482, *p* = 2.36e-3), macrophages (Rho = 0.598, *p* = 1.79e-51), and neutrophils (Rho = 0.514, *p* = 2.79e-36) (Figure 4D). In BRCA-LumB (*n* = 219), INHBA expression had significantly negative correlation with NKT cell infiltration level (Rho = -0.331, *p* = 2.74e-6) and showed positive correlations with infiltrating levels of CD8^+^ T cells (Rho = 0.218, *p* = 2.41e-3), macrophages (Rho = 0.624, *p* = 4.10e-22), and neutrophils (Rho = 0.452, *p* = 4.91e-11) (Figure 4E). Most strikingly, INHBA expression was strongly associated with the CAF infiltrations in BRCA (Rho = 0.718, *p* = 1.29e-158), BRCA-Basal (Rho = 0.635, *p* = 4.17e-21), BRCA-Her2 (Rho = 0.802, *p* = 1.6e-17), BRCA-LumA (Rho = 0.736, *p* = 2.46e-89), and BRCA-LumB (Rho = 0.801, *p* = 1.61e-44) subtypes (Figure 5). Taken together, these results indicate that INHBA plays an important role in regulating the infiltration of immune cells, especially macrophages and CAFs, in the tumor microenvironment of breast cancer.

### Correlation Analysis Between INHBA and Infiltrating Immune Cells Markers Expression in Breast Cancer

To further investigate the effects of INHBA expression on infiltration levels of immune cells, we next performed correlation analysis between INHBA expression and related markers of infiltrating immune cells in BRCA and the subtypes (BRCA-Basal, BRCA-Her2, BRCA-LumA, and BRCA-LumB) by TIMER2.0. First, we analyzed the correlations between INHBA and specific markers of innate immune cells (monocytes, TAMs, M1 and M2 macrophages, neutrophils, NK cells, and DCs) (Supplementary Table S2), adaptive immune cells (CD8^+^ T cells, general T cells, B cells, Th1 cells, Th2 cells, Tfh cells, Th17 cells, Th9 cells, Th22, Tregs, and exhausted T cells) (Supplementary Table S3), and CAFs (Table 3).

**TABLE 3:** Correlation analysis between INHBA and related genes and markers of CAFs in TIMER2.0.

Description	Gene marker	BRCA		BRCA-Basal		BRCA-Her2		BRCA-LumA		BRCA-LumB	
		Rho	*P*	Rho	*P*	Rho	*P*	Rho	*P*	Rho	*P*
CAF	Vimentin (VIM)	0.397	***	0.331	***	0.607	***	0.504	***	0.560	***
	α-SMA (ACTA2)	0.455	***	0.405	***	0.644	***	0.490	***	0.620	***
	FAP	0.731	***	0.616	***	0.839	***	0.755	***	0.745	***
	Periostin (POSTN)	0.743	***	0.640	***	0.796	***	0.785	***	0.779	***
	PDGFRA	0.583	***	0.395	***	0.754	***	0.673	***	0.723	***
	PDGFRB	0.704	***	0.613	***	0.841	***	0.780	***	0.721	***
	THY1	0.588	***	0.451	***	0.621	***	0.624	***	0.578	***
	Podoplanin (PDPN)	0.627	***	0.629	***	0.816	***	0.626	***	0.661	***
	Integrin β1(ITGB1)	0.702	***	0.565	***	0.860	***	0.789	***	0.637	***
	Nidogen-2 (NID2)	0.788	***	0.651	***	0.815	***	0.833	***	0.741	***

BRCA, breast invasive carcinoma; CAF, cancer-associated fibroblast; Purity, correlation adjusted for tumor purity; Cor, R value of Spearman's correlation. *p < 0.01; **p < 0.001; ***p < 0.0001.

In Supplementary Table S2, the results after adjustment for purity showed that INHBA expression level was significantly related to most immune markers of innate immune cells in BRCA and the subtypes. Most interestingly, we found that INHBA expression level was highly related to almost all common markers of monocytes/macrophages (monocytes, TAMs, and M1 and M2 macrophages) in BRCA and the subtypes. Then, we further validated the relationship between INHBA and the markers discussed earlier of monocytes, TAMs, and M1 and M2 macrophages in normal tissues and BRCA by GEPIA2. Consistent with the results of the TIMER2.0 analysis, INHBA expression was also highly correlated with those immune markers in BRCA (Table 4). These results reveal that INHBA may play an important role in regulating macrophage polarization in BRCA. Consistent with the results of correlations between INHBA expression and infiltrating level of neutrophils, INHBA expression level was also significantly associated with the neutrophil marker CD15 in BRCA, BRCA-LumA, and BRCA-LumB subtypes. In addition, INHBA expression level was significantly related to the DC markers (HLA-DPA, HLA-DPA1, BDCA-4, CD11c, and CD141) in BRCA, BRCA-LumA, and BRCA-LumB subtypes. In Supplementary Table S3 **,** we observed that there were strong correlations between INHBA and Tregs markers (FOXP3, CCR8, CD25, STAT5B, and TGFβ) in BRCA and BRCA-Luminal subtype. These findings indicate that INHBA is indeed involved in immune infiltrations in breast cancer.

**TABLE 4:** Correlation analysis between INHBA and related genes and markers of monocytes/macrophages and CAFs in GEPIA2.

Description	Gene marker	BRCA			
		Normal		Tumor	
		R	*P*	R	*P*
Monocyte	CD115(CSF1R)	0.170	0.078	0.310	***
	CD16(FCGR3A)	0.290	*	0.490	***
	CD86	0.330	**	0.370	***
TAM	CD11b (ITGAM)	0.280	*	0.340	***
	CCL2	0.330	**	0.210	***
	CD68	0.230	0.017	0.410	***
	CD80	0.560	***	0.410	***
M1 Macrophage	iNOS (NOS2)	0.031	0.75	0.370	***
	IRF5	0.260	*	0.092	*
	COX2 (PTGS2)	0.390	***	0.240	***
	CXCL10	0.330	**	0.170	***
	ROS1	0.240	0.011	0.360	***
	HLA-DRA	0.280	*	0.260	***
M2 Macrophage	CD163	0.100	0.290	0.180	***
	VSIG4	0.120	0.200	0.250	***
	MS4A4A	0.170	0.075	0.270	***
	CD206 (MRC1)	0.200	0.035	0.250	***
	CD209	0.170	0.077	0.180	***
CAF	Vimentin (VIM)	0.016	0.870	0.450	***
	α-SMA (ACTA2)	0.270	*	0.540	***
	FAP	0.350	**	0.760	***
	Periostin (POSTN)	0.160	0.100	0.780	***
	PDGFRA	0.460	***	0.600	***
	PDGFRB	0.270	*	0.730	***
	THY1	0.300	*	0.760	***
	Podoplanin (PDPN)	0.480	***	0.690	***
	Integrin β1 (ITGB1)	0.220	0.019	0.710	***
	Nidogen-2 (NID2)	0.220	0.022	0.780	***

BRCA, breast invasive carcinoma; TAM, tumor-associated macrophage; CAF, cancer-associated fibroblast; Tumor, correlation analysis in tumor tissue of TCGA. Normal, correlation analysis in normal mammary tissue of TCGA. *p < 0.01; **p < 0.001; ***p < 0.0001.

In Table 3, TIMER2.0 analysis showed that the expression level of INHBA was highly correlated with the expression of common markers of CAFs. Among them, *α*-SMA (ACTA2), FAP, Periostin (POSTN), PDGFRA, PDGFRB, THY1, Podoplanin (PDPN), and Integrinβ1 (ITGB1) were activated CAF markers (Han et al., 2020; Kanzaki and Pietras, 2020). Consistent with the results of the TIMER2.0 analysis, INHBA expression also had strong correlations with those immune markers in BRCA by GEPIA2 (Table 4). These results suggest that INHBA also plays an important role in increasing the infiltration of CAFs in the tumor microenvironment of breast cancer.

## Discussion

In Global Cancer Statistics 2020, breast cancer was reported as the most commonly diagnosed cancer and the leading cause of cancer death among women worldwide (Sung et al., 2021). Breast cancer is a complex and heterogeneous disease (Harbeck and Gnant, 2017). Furthermore, the immune microenvironment influences breast cancer development and outcome by promoting tumor immune escape (Tekpli et al., 2019). It is crucial to identify novel biomarkers which have prognostic, predictive, and therapeutic roles in breast cancer.

TGF-β signaling contributes to cancer progression by promoting metastasis and suppressing the antitumor activities of immune cells (Derynck et al., 2021). Nevertheless, INHBA, as a member of the TGF-β family, has pro- or antitumorigenic effects in diverse cancers. For instance, high INHBA expression promoted malignant biological behavior by activating the TGF-β pathway in colorectal cancer and predicted the patients' prognosis (Okano et al., 2013; He et al., 2021; Li X. et al., 2020). In contrast, INHBA was significantly downregulated and functioned as a tumor suppressor in diffuse large B-cell lymphoma (Jiang et al., 2018). Of interest, INHBA was involved in the progression of ductal carcinoma *in situ* to invasive breast cancer (Liu et al., 2019). The INHBA expression level could also serve as predictors of treatment effect and prognosis of patients with early breast cancer (Wang et al., 2020). However, the significance of INHBA expression in the prognosis of patients with breast cancer remains unclear. Here, we first identify INHBA as a potential biomarker of prognosis and tumor-infiltrating immune cells.

In this study, we first assessed the expression profile of INHBA in cancers using Oncomine and TIMER2.0 databases. Oncomine data showed that INHBA mRNA expression obviously increased in multiple cancers compared with normal tissue and particularly in breast cancer. TCGA data determined by TIMER2.0 revealed that INHBA was highly expressed in BRCA and BRCA subtypes (BRCA-Basal, BRCA-HER2, and BRCA-LumA and BRCA-LumB) relative to normal adjacent tissues. Meanwhile, the UALCAN cancer database showed that INHBA protein was also overexpressed in primary breast cancer compared with normal tissues. Meanwhile, the methylation level of the INHBA gene promoter was significantly reduced in breast cancer (data not shown), suggesting that the upregulation of INHBA gene expression in breast cancer may be related to the decreased methylation level of the INHBA gene promoter. The bc-GenExMiner v4.7 database indicated that progesterone receptor, human epidermal growth factor receptor-2 status, and PAM50 subtypes were positively correlated with INHBA expression. Conversely, age and basal-like status were negatively related to INHBA level in breast cancer samples with respect to normal tissues. However, the UALCAN cancer database showed that INHBA protein expression was significantly higher in the stage I group compared with the stage II and III groups. Its protein expression was not related to major subclass, tumor histology, patient's race, and age. We found that the sample size of INHBA protein expression was small (normal *n* = 18; breast cancer *n* = 125), and more proof was needed. Thus, these findings indicated that INHBA expression might predict the prognosis of breast cancer. Next, we further explored the prognostic value of INHBA in breast cancer using the PrognoScan and Kaplan–Meier plotter database. Breast cancer patients with increased INHBA demonstrated worse OS, RFS, and DMFS. In addition, a high level of INHBA expression was significantly correlated with worse OS and RFS in breast cancer with positive lymph nodes. Together, these results strongly suggested that INHBA is a novel prognostic-related biomarker in breast cancer.

Interestingly, we also found that INHBA expression was associated with the level of immune infiltration in breast cancer. By analyzing the TIMER2.0 database, we observed that INHBA expression was significantly negatively correlated with infiltration levels of activated NK, NKT, and CD4^+^ cells and positively correlated with infiltration level of neutrophils in BRCA and almost all subtypes. NK, NKT, and CD4^+^ cells show cytotoxicity against various tumor cells, including breast cancer. They can be inhibited and promote tumor progression (Gonzalez-Navajas et al., 2021; Liu et al., 2021). Tumor-associated neutrophils were reported to participate in tumor promotion and development by induction of immunosuppression in breast cancer (Hajizadeh et al., 2021). Thus, INHBA may play a critical role in inhibiting antitumor immune response. The TIMER2.0 and GEPIA2 analysis also showed that INHBA expression had significant correlations with monocyte/macrophage infiltrations in BRCA and almost all subtypes. These results suggest that INHBA may affect TAM function and participate in the progression of breast cancer by regulating M1/M2 polarization of macrophages. In addition, INHBA expression level was also significantly associated with the DC markers and Tregs markers in BRCA, BRCA-LumA, and BRCA-LumB subtypes. In particular, FOXP3 plays a key role in Tregs development and function. However, too many Tregs could prevent the immune system from destroying tumor cells and promoting tumor progression (Tanaka and Sakaguchi, 2019). In addition, studies have found that DCs could promote tumor metastasis by enhancing Tregs responses and suppressing CD8^+^ T cell cytotoxicity (Sawant et al., 2012; Solis-Castillo et al., 2020). INBHA may promote Tregs and DC response to prevent T cell-mediated immunity from destroying tumor cells. Besides, we also found that INHBA was highly correlated with the abundance of CAFs. As is known to all, CAFs are crucial components in the tumor microenvironment, play pivotal roles in tumor progression, and further impact clinical prognosis (Giorello et al., 2021). In breast cancer, CAFs can interact with tumor-infiltrating immune cells and promote tumor development and immunosuppression (Gunaydin, 2021; Soongsathitanon et al., 2021). Together, these findings indicate that INHBA may potentially inhibit the immune response by regulating immune cell recruitment and activation in breast cancer. Of course, there are also some limitations in our study. We explore the clinical relevance of the INHBA gene just by utilizing various publicly available resources and databases; there still needs further verification and support through much more experiments in future studies.

In summary, INHBA is an important regulator of immune cell infiltration and a valuable prognostic biomarker in breast cancer patients. Further studies are warranted to clarify the significance of INHBA in breast cancer treatment.

## Data Availability

The datasets presented in this study can be found in online repositories. The names of the repository/repositories and accession number(s) can be found in the article/Supplementary Material.
