# Corrigendum: Increased Expression of INHBA Is Correlated With Poor Prognosis and High Immune Infiltrating Level in Breast Cancer

**DOI:** 10.3389/fbinf.2022.974000

**Published:** 2022-07-20

**Authors:** Zeying Yu, Li Cheng, Xinlian Liu, Lushun Zhang, Hui Cao

**Affiliations:** ^1^ Department of Pathogenic Biology, School of Basic Medical Science, Chengdu Medical College, Chengdu, China; ^2^ School of Basic Medical Science, Chengdu Medical College, Chengdu, China; ^3^ Department of Pathology and Pathophysiology, School of Basic Medical Science, Chengdu Medical College, Chengdu, China

**Keywords:** breast cancer, macrophages, prognosis, INHBA, CAFs

In the published article, there was an error in the **Funding** statement. The funding number for the Research Foundation of Chengdu Medical College was displayed as “No. 16Z141.” The correct number is “No. CYZ16-02.” The correct **Funding** statement appears below:

“This study was supported by grants from the Research Foundation of Chengdu Medical College (No. CYZ16-02) and Sichuan Science and Technology Foundation (No. 2021YJ0089).”

The authors apologize for this error and state that this does not change the scientific conclusions of the article in any way. The original article has been updated.

