# Supplementary material for: Increased Expression of INHBA Is Correlated With Poor Prognosis and High Immune Infiltrating Level in Breast Cancer
Source: Front Bioinform. 2022 Mar 24;2:729902. doi: 10.3389/fbinf.2022.729902 (PMC9580937; doi:10.3389/fbinf.2022.729902)
Supplement: Supplementary file 1 [file DataSheet1.docx]

**Supplementary materials**

**Supplementary Table 1.** INHBA mRNA expression in different subtypes of breast cancer and normal breast tissues (Oncomine database)

| **Reference** | **Subtype of breast cancer vs. normal** | ***P*-value** | ***t* test** | **Fold change** | **Gene rank** | **Sample** |
| --- | --- | --- | --- | --- | --- | --- |
| Ma Breast 4 Statistics | Ductal breast carcinoma *in situ* | 8.21E-11 | 12.754 | 21.042 | 43 | 11 |
| Ma Breast 4 Statistics | Invasive ductal breast carcinoma | 8.33E-6 | 7.666 | 21.125 | 5 | 9 |
| Karnoub Breast Statistics | Invasive ductal breast carcinoma | 1.17E-4 | 4.716 | 10.445 | 242 | 7 |
| Turashvili Breast Statistics | Invasive ductal breast carcinoma | 2.00E-5 | 5.134 | 9.755 | 16 | 5 |
| TCGA Breast Statistics | Mixed lobular and ductal breast carcinoma | 5.28E-6 | 10.160 | 9.633 | 285 | 7 |
| TCGA Breast Statistics | Invasive ductal and lobular carcinoma | 6.35E-4 | 12.207 | 8.827 | 1170 | 3 |
| TCGA Breast Statistics | Invasive lobular breast carcinoma | 2.31E-24 | 15.766 | 8.384 | 10 | 36 |
| TCGA Breast Statistics | Invasive breast carcinoma | 1.22E-44 | 20.989 | 8.351 | 6 | 76 |
| TCGA Breast Statistics | Invasive ductal breast carcinoma | 5.83E-48 | 26.769 | 7.891 | 61 | 389 |
| TCGA Breast Statistics | Mucinous breast carcinoma | 9.92E-6 | 12.098 | 4.501 | 234 | 4 |
| Richardson Breast 2 Statistics | Ductal breast carcinoma | 2.04E-12 | 11.125 | 8.946 | 51 | 40 |
| Sorlie Breast Statistics | Ductal breast carcinoma | 4.81E-7 | 11.442 | 6.459 | 51 | 64 |
| Sorlie Breast 2 Statistics | Ductal breast carcinoma | 6.43E-5 | 10.571 | 6.331 | 149 | 90 |
| Perou Breast Statistics | Lobular breast carcinoma | 9.95E-4 | 6.137 | 5.825 | 11 | 4 |
| Perou Breast Statistics | Ductal breast carcinoma | 3.62E-4 | 8.398 | 4.875 | 211 | 36 |
| Zhao Breast Statistics | Lobular breast carcinoma | 1.27E-4 | 8.618 | 5.339 | 783 | 20 |
| Radvanyi Breast Statistics | Invasive mixed breast carcinoma | 3.53E-4 | 6.808 | 3.909 | 35 | 3 |
| Radvanyi Breast Statistics | Invasive lobular breast carcinoma | 9.90E-4 | 4.568 | 2.765 | 41 | 6 |
| Radvanyi Breast Statistics | Invasive ductal breast carcinoma | 8.54E-4 | 4.299 | 2.705 | 127 | 31 |

**Supplementary Table 2.** Correlation analysis between INHBA and related genes and markers of innate immune cells in TIMER2.0.

| **Description** | **Gene marker** | **BRCA** | |  | **BRCA-Basal** | |  | **BRCA-Her2** | |  | **BRCA-LumA** | |  | **BRCA-LumB** | |
| --- | --- | --- | --- | --- | --- | --- | --- | --- | --- | --- | --- | --- | --- | --- | --- |
|  |  | **Rho** | ***P*** |  | **Rho** | ***P*** |  | **Rho** | ***P*** |  | **Rho** | ***P*** |  | **Rho** | ***P*** |
| Monocyte | CD115(CSF1R) | 0.297 | *** |  | 0.234 | * |  | 0.434 | ** |  | 0.308 | *** |  | 0.363 | *** |
|  | CD16(FCGR3A) | 0.474 | *** |  | 0.338 | *** |  | 0.598 | *** |  | 0.498 | *** |  | 0.497 | *** |
|  | CD86 | 0.338 | *** |  | 0.205 | * |  | 0.385 | ** |  | 0.378 | *** |  | 0.358 | *** |
| TAM | CD11b(ITGAM) | 0.323 | *** |  | 0.292 | *** |  | 0.334 | * |  | 0.326 | *** |  | 0.324 | *** |
|  | CCL2 | 0.167 | *** |  | 0.220 | * |  | 0.167 | 0.160 |  | 0.184 | *** |  | 0.164 | 0.023 |
|  | CD68 | 0.374 | *** |  | 0.263 | ** |  | 0.403 | ** |  | 0.396 | *** |  | 0.376 | *** |
|  | CD80 | 0.420 | *** |  | 0.299 | *** |  | 0.513 | *** |  | 0.492 | *** |  | 0.438 | *** |
| M1 | iNOS(NOS2) | 0.345 | *** |  | 0.299 | *** |  | 0.535 | *** |  | 0.388 | *** |  | 0.366 | *** |
|  | IRF5 | 0.073 | 0.022 |  | 0.114 | 0.134 |  | 0.170 | 0.154 |  | 0.072 | 0.101 |  | -0.017 | 0.810 |
|  | COX2(PTGS2) | 0.179 | *** |  | 0.246 | * |  | 0.230 | 0.052 |  | 0.249 | *** |  | 0.290 | *** |
|  | CXCL10 | 0.155 | *** |  | 0.014 | 0.852 |  | 0.087 | 0.469 |  | 0.215 | *** |  | 0.212 | * |
|  | ROS1 | 0.398 | *** |  | 0.275 | ** |  | 0.466 | *** |  | 0.520 | *** |  | 0.415 | *** |
|  | HLA-DRA | 0.234 | *** |  | 0.090 | 0.237 |  | 0.021 | 0.862 |  | 0.312 | *** |  | 0.248 | ** |
| M2 | CD163 | 0.306 | *** |  | 0.306 | *** |  | 0.405 | ** |  | 0.314 | *** |  | 0.329 | *** |
|  | VSIG4 | 0.246 | *** |  | 0.207 | * |  | 0.439 | ** |  | 0.244 | *** |  | 0.288 | *** |
|  | MS4A4A | 0.248 | *** |  | 0.235 | * |  | 0.309 | * |  | 0.258 | *** |  | 0.238 | ** |
|  | CD206(MRC1) | 0.222 | *** |  | 0.299 | *** |  | 0.190 | 0.111 |  | 0.277 | *** |  | 0.177 | 0.106 |
|  | CD209 | 0.137 | *** |  | 0.180 | 0.017 |  | 0.190 | 0.111 |  | 0.200 | *** |  | 0.006 | 0.932 |
| Neutrophils | CD66b(CEACAM8) | -0.042 | 0.183 |  | 0.054 | 0.477 |  | -0.061 | 0.609 |  | -0.047 | 0.290 |  | -0.116 | 0.109 |
|  | CCR7 | -0.076 | 0.163 |  | -0.007 | 0.931 |  | -0.316 | * |  | -0.056 | 0.206 |  | -0.110 | 0.128 |
|  | CD15(FUT4) | 0.258 | *** |  | 0.176 | 0.020 |  | 0.148 | 0.214 |  | 0.402 | *** |  | 0.375 | *** |
|  | MPO | -0.002 | 0.937 |  | 0.069 | 0.365 |  | -0.071 | 0.553 |  | 0.058 | 0.187 |  | 0.057 | 0.432 |
| NK cell | KIR2DL1 | -0.042 | 0.182 |  | -0.173 | 0.022 |  | -0.158 | 0.186 |  | -0.014 | 0.747 |  | 0.003 | 0.969 |
|  | KIR2DL3 | 0.030 | 0.345 |  | 0.006 | 0.934 |  | -0.195 | 0.100 |  | 0.055 | 0.211 |  | 0.053 | 0.461 |
|  | KIR2DL4 | -0.053 | 0.097 |  | -0.131 | 0.086 |  | -0.248 | 0.036 |  | -0.029 | 0.516 |  | -0.054 | 0.454 |
|  | KIR3DL1 | 0.011 | 0.735 |  | -0.056 | 0.461 |  | -0.014 | 0.908 |  | 0.023 | 0.605 |  | 0.020 | 0.780 |
|  | KIR3DL2 | -0.024 | 0.459 |  | -0.069 | 0.363 |  | -0.315 | * |  | 0.046 | 0.302 |  | -0.063 | 0.383 |
|  | KIR3DL3 | -0.035 | 0.269 |  | -0.046 | 0.549 |  | -0.138 | 0.248 |  | -0.012 | 0.793 |  | -0.028 | 0.700 |
|  | KIR2DS4 | -0.001 | 0.982 |  | -0.058 | 0.449 |  | -0.118 | 0.325 |  | 0.045 | 0.310 |  | -0.061 | 0.399 |
|  | XCL1 | -0.039 | 0.214 |  | -0.111 | 0.144 |  | -0.157 | 0.187 |  | 0.049 | 0.262 |  | -0.076 | 0.297 |
|  | CD7 | -0.138 | *** |  | -0.198 | * |  | -0.312 | * |  | -0.109 | 0.013 |  | -0.059 | 0.418 |
| DC | HLA-DPB1 | 0.058 | 0.066 |  | -0.018 | 0.818 |  | -0.106 | 0.376 |  | 0.088 | 0.045 |  | 0.116 | 0.108 |
|  | HLA-DQB1 | 0.065 | 0.041 |  | -0.016 | 0.831 |  | -0.039 | 0.746 |  | 0.096 | 0.029 |  | 0.029 | 0.688 |
|  | HLA-DRA | 0.234 | *** |  | 0.090 | 0.237 |  | 0.021 | 0.862 |  | 0.312 | *** |  | 0.248 | ** |
|  | HLA-DPA1 | 0.204 | *** |  | 0.043 | 0.577 |  | -0.033 | 0.780 |  | 0.279 | *** |  | 0.230 | * |
|  | BDCA-4(NRP1) | 0.585 | *** |  | 0.451 | *** |  | 0.687 | *** |  | 0.631 | *** |  | 0.635 | *** |
|  | CD11C(ITGAX) | 0.320 | *** |  | 0.292 | *** |  | 0.310 | * |  | 0.314 | *** |  | 0.318 | *** |
|  | NKp46(NCR1) | 0.065 | 0.041 |  | -0.022 | 0.774 |  | 0.020 | 0.868 |  | 0.092 | 0.037 |  | 0.079 | 0.275 |
|  | CD141(THBD) | 0.244 | *** |  | 0.386 | *** |  | 0.349 | * |  | 0.193 | *** |  | 0.251 | ** |

BRCA, breast invasive carcinoma; TAM, tumor-associated macrophage; NK cell, natural killer cell; DC, dendritic cell; Cor, R value of Spearman’s correlation adjusted for tumor purity. **P* < 0.01; ***P* < 0.001;****P* < 0.0001.

**Supplementary Table 3.** Correlation analysis between INHBA and related genes and markers of adaptive immune cells in TIMER 2.0.

| **Description** | **Gene marker** | **BRCA** | |  | **BRCA-Basal** | |  | **BRCA-Her2** | |  | **BRCA-LumA** | |  | **BRCA-LumB** | |
| --- | --- | --- | --- | --- | --- | --- | --- | --- | --- | --- | --- | --- | --- | --- | --- |
|  |  | **Rho** | ***P*** |  | **Rho** | ***P*** |  | **Rho** | ***P*** |  | **Rho** | ***P*** |  | **Rho** | ***P*** |
| CD8+ T cell | CD8A | -0.015 | 0.635 |  | -0.092 | 0.229 |  | -0.202 | 0.089 |  | 0.065 | 0.140 |  | -0.076 | 0.297 |
|  | CD8B | -0.099 | * |  | -0.143 | 0.059 |  | -0.191 | 0.108 |  | -0.038 | 0.385 |  | -0.062 | 0.395 |
| T cell (general) | CD3D | -0.057 | 0.071 |  | -0.134 | 0.078 |  | -0.274 | 0.020 |  | -0.007 | 0.875 |  | -0.038 | 0.605 |
|  | CD3E | -0.013 | 0.674 |  | -0.074 | 0.331 |  | -0.283 | 0.016 |  | 0.044 | 0.316 |  | 0.001 | 0.990 |
|  | CD2 | 0.029 | 0.361 |  | -0.042 | 0.586 |  | -0.214 | 0.071 |  | 0.085 | 0.053 |  | 0.041 | 0.570 |
| B cell | CD19 | -0.113 | ** |  | -0.054 | 0.483 |  | -0.385 | ** |  | -0.097 | 0.028 |  | -0.198 | * |
|  | CD138(SDC1) | 0.466 | *** |  | 0.234 | * |  | 0.600 | *** |  | 0.586 | *** |  | 0.410 | *** |
|  | CD79A | -0.061 | 0.055 |  | 0.004 | 0.959 |  | -0.239 | 0.043 |  | -0.060 | 0.174 |  | -0.103 | 0.156 |
|  | CD20(MS4A1) | -0.051 | 0.106 |  | 0.047 | 0.534 |  | -0.361 | * |  | -0.010 | 0.819 |  | -0.189 | * |
|  | CD38 | 0.027 | 0.390 |  | -0.046 | 0.546 |  | -0.118 | 0.324 |  | 0.068 | 0.124 |  | 0.045 | 0.539 |
|  | CD23(FCER2) | -0.149 | *** |  | -0.061 | 0.424 |  | -0.334 | * |  | -0.112 | 0.010 |  | -0.215 | * |
| Th1 | T-bet(TBX21) | -0.068 | 0.031 |  | -0.114 | 0.136 |  | -0.278 | 0.018 |  | -0.041 | 0.357 |  | -0.027 | 0.707 |
|  | STAT4 | 0.122 | ** |  | 0.113 | 0.137 |  | -0.067 | 0.577 |  | 0.173 | *** |  | 0.098 | 0.175 |
|  | STAT1 | 0.267 | *** |  | 0.192 | 0.011 |  | 0.160 | 0.178 |  | 0.328 | *** |  | 0.228 | * |
|  | IFN-γ(IFNG) | -0.001 | 0.970 |  | -0.072 | 0.342 |  | -0.128 | 0.286 |  | 0.029 | 0.514 |  | -0.007 | 0.922 |
|  | IL12RB2 | 0.083 | * |  | 0.009 | 0.911 |  | -0.004 | 0.975 |  | 0.188 | *** |  | 0.217 | * |
|  | WSX-1(IL27RA) | -0.081 | 0.011 |  | -0.104 | 0.172 |  | -0.131 | 0.272 |  | 0.002 | 0.968 |  | -0.036 | 0.620 |
|  | T-BET(TBX21) | -0.068 | 0.031 |  | -0.114 | 0.136 |  | -0.278 | 0.018 |  | -0.041 | 0.357 |  | -0.027 | 0.707 |
| Th2 | GATA3 | 0.066 | 0.037 |  | -0.020 | 0.794 |  | -0.005 | 0.964 |  | 0.003 | 0.939 |  | -0.004 | 0.954 |
|  | STAT6 | 0.105 | ** |  | 0.073 | 0.338 |  | 0.159 | 0.182 |  | 0.121 | * |  | 0.112 | 0.122 |
|  | STAT5A | 0..051 | 0.111 |  | 0.043 | 0.570 |  | 0.134 | 0.262 |  | 0.112 | 0.010 |  | 0.052 | 0.478 |
|  | IL13 | 0.021 | 0.516 |  | -0.021 | 0.516 |  | -0.026 | 0.826 |  | 0.033 | 0.457 |  | 0.037 | 0.612 |
|  | CCR3 | 0.104 | * |  | 0.071 | 0.350 |  | 0.009 | 0.941 |  | 0.135 | * |  | 0.085 | 0.241 |
| Tfh | BCL6 | 0.183 | *** |  | 0.199 | * |  | 0.114 | 0.340 |  | 0.209 | *** |  | 0.163 | 0.023 |
|  | IL21 | 0.013 | 0.676 |  | 0.011 | 0.886 |  | -0.297 | 0.011 |  | 0.066 | 0.136 |  | -0.018 | 0.804 |
|  | CXCR5 | -0.058 | 0.065 |  | -0.012 | 0.874 |  | -0.270 | 0.022 |  | -0.043 | 0.334 |  | -0.128 | 0.078 |
|  | CD278(ICOS) | 0.123 | ** |  | 0.060 | 0.430 |  | 0.018 | 0.878 |  | 0.170 | *** |  | 0.163 | 0.024 |
|  | CXCL13 | -0.042 | 0.191 |  | -0.063 | 0.411 |  | -0.161 | 0.177 |  | -0.020 | 0.647 |  | 0.004 | 0.961 |
| Th17 | STAT3 | 0.376 | *** |  | 0.392 | *** |  | 0.429 | ** |  | 0.457 | *** |  | 0.258 | ** |
|  | IL17A | 0.027 | 0.396 |  | 0.054 | 0.483 |  | 0.051 | 0.672 |  | 0.012 | 0.785 |  | 0.098 | 0.177 |
|  | IL21R | 0.326 | *** |  | 0.125 | 0.101 |  | 0.141 | 0.236 |  | 0.445 | *** |  | 0.331 | *** |
|  | IL23R | 0.127 | *** |  | 0.239 | * |  | 0.012 | 0.917 |  | 0.180 | *** |  | 0.051 | 0.485 |
| Th9 | TGFBR2 | 0.503 | *** |  | 0.476 | *** |  | 0.575 | *** |  | 0.529 | *** |  | 0.610 | *** |
|  | IRF4 | 0.085 | * |  | 0.051 | 0.502 |  | -0.143 | 0.231 |  | 0.143 | * |  | 0.088 | 0.223 |
|  | PU.1(SPI1) | 0.142 | *** |  | 0.022 | 0.778 |  | 0.139 | 0.244 |  | 0.152 | ** |  | 0.205 | * |
| Th22 | CCR10 | -0.052 | 0.100 |  | 0.066 | 0.386 |  | -0.019 | 0.875 |  | -0.117 | * |  | -0.013 | 0.860 |
|  | AHR | 0.398 | *** |  | 0.442 | *** |  | 0.400 | ** |  | 0.433 | *** |  | 0.358 | *** |
| Treg | FOXP3 | 0.196 | *** |  | 0.100 | 0.189 |  | 0.097 | 0.420 |  | 0.253 | *** |  | 0.190 | * |
|  | CCR8 | 0.349 | *** |  | 0.348 | *** |  | 0.342 | * |  | 0.387 | *** |  | 0.315 | *** |
|  | CD25(IL2RA) | 0.241 | *** |  | 0.218 | * |  | 0.227 | 0.055 |  | 0.334 | *** |  | 0.274 | ** |
|  | STAT5B | 0.175 | *** |  | 0.226 | * |  | 0.316 | * |  | 0.241 | *** |  | 0.031 | 0.666 |
|  | TGFβ(TGFB1) | 0.340 | *** |  | 0.318 | *** |  | 0.479 | *** |  | 0.309 | *** |  | 0.433 | *** |
| T cell exhaustion | PD-1(PDCD1) | -0.096 | * |  | -0.135 | 0.075 |  | -0.205 | 0.084 |  | -0.072 | 0.104 |  | -0.102 | 0.159 |
|  | CTLA4 | 0.057 | 0.072 |  | -0.019 | 0.803 |  | -0.100 | 0.401 |  | 0.095 | 0.031 |  | 0.099 | 0.171 |
|  | LAG3 | -0.098 | * |  | -0.185 | 0.014 |  | -0.142 | 0.234 |  | -0.096 | 0.029 |  | -0.052 | 0.471 |
|  | TIM-3(HAVCR2) | 0.415 | *** |  | 0.234 | * |  | 0.514 | *** |  | 0.453 | *** |  | 0.439 | *** |
|  | GZMB | -0.059 | 0.064 |  | -0.095 | 0.214 |  | -0.366 | * |  | 0.006 | 0.890 |  | -0.060 | 0.405 |

BRCA, breast invasive carcinoma; Th, T helper cell; Tfh, follicular T cell; Treg, regulatory T call; Cor, R value of Spearman’s correlation adjusted for tumor purity. **P*<0.01; ***P*<0.001;****P*<0.0001.
